# Role of NP-59 SPECT/CT in the Management of Mild Autonomous Cortisol Secretion

**DOI:** 10.3390/jcm15166302

**Published:** 2026-08-14

**Authors:** Łukasz Żukowski, Michał Szklarz, Dariusz Piechocki, Piotr Michał Szumowski, Małgorzata Mojsak, Saeid Abdelrazek, Izabela Sulima, Wojciech Matuszewski, Janusz Myśliwiec

**Affiliations:** 1 Department of Nuclear Medicine, Medical University of Bialystok, M. Skłodowskiej-Curie St. 24A, 15-276 Bialystok, Poland; 2 Clinic of Endocrinology, Diabetology and Internal Medicine, University of Warmia and Mazury in Olsztyn, 10-957 Olsztyn, Poland

**Keywords:** NP-59, mild autonomous cortisol secretion, SPECT/CT, comorbidities

## Abstract

**Background/Objectives**: Recent advancements in medical imaging have provided tools that significantly enhance diagnostic precision and patient care. Among these, NP-59 SPECT/CT imaging has emerged as a promising method for assessing adrenal gland functionality. Current gaps in clinical practice, including the lack of specific diagnostic biomarkers and the paucity of clinical trials demonstrating the efficacy of adrenalectomy in alleviating comorbidities, necessitate the search for other diagnostic methods. Management becomes more challenging when bilateral adrenal adenomas are present. Demonstrating lateralization in cortisol secretion may greatly assist in treatment selection. Unilateral secretion of cortisol indicates autonomy and therefore adrenalectomy should be preferred. The aim of this study was to evaluate the usefulness of NP-59 SPECT/CT in the management of MACS. **Methods**: We investigated 20 patients with benign bilateral adrenal incidentalomas and MACS between the years 2023 and 2025. The first group (70%) consisted of patients with only hypertension, without additional comorbidities (hypertension only, HO). The second group (30%) consisted of hypertensive patients with additional comorbidities (hypertension with comorbidities, HC). Visualizations of adrenal glands metabolic activity were conducted using the SPECT/CT system after intravenous injection of NP-59. Abdominal SPECT/CT was performed at 3, 5, and 7 days after tracer injection. Lateralization was established when only single adrenal uptake occurred as early as the third day. **Results**: Six (30%) patients demonstrated lateralization during NP-59 SPECT scintigraphy. All these patients belonged to the HO group and accounted for 43% of it. **Conclusions**: NP-59 SPECT scintigraphy is valuable in the identification of unilateral cortisol secretion and guiding treatment decisions such as adrenalectomy versus conservative management. Prospective studies are needed to determine its clinical utility.

## 1. Introduction

In mild autonomous cortisol secretion (MACS), the adrenal glands produce cortisol without the usual regulation from the hypothalamus–pituitary–adrenal (HPA) axis. This condition is often identified in individuals with adrenal incidentalomas and unlike overt Cushing’s syndrome, the signs and symptoms of cortisol excess in MACS may be subtle or absent. Prevalence among adrenal incidentalomas ranges from 20 to 50% [1,2,3,4] depending on the different criteria used in different studies. The overall prevalence of adrenal masses is approximately 2% and increases with age [1,2,5,6]. A population-based study showed that the incidence of detected adrenal tumors increased tenfold between 1995 and 2017 due to the increasing number of abdominal imaging examinations [1]. According to Endocrine Society, MACS is defined as serum cortisol levels > 1.8 µg/dL (>50 nmol/L) after the 1 mg dexamethasone suppression test (DST) [7,8,9,10]. MACS, similar to overt Cushing’s syndrome, is also associated with increased mortality and decreased quality of life [11]. Treatment for MACS should be tailored to individual patient characteristics and includes adrenalectomy or conservative treatment along with treatment of comorbidities. Difficulties in clinical practice are caused by the lack of specific diagnostic biomarkers. Furthermore, the results of clinical trials demonstrating the effectiveness of adrenalectomy in improving comorbidities are inconclusive, as this benefit is not always observed in all patients with MACS [11]. There is little evidence that long-term conservative treatment in patients with MACS is effective and safe [12]. Identifying patients who seem to benefit from adrenalectomy is challenging. MACS occurs more frequently in bilateral adrenal incidentalomas [13,14] than in unilateral lesions, which makes its management even more difficult. Demonstrating lateralization in cortisol secretion should greatly assist in treatment selection. Unilateral secretion of cortisol indicates autonomy and therefore adrenalectomy is preferred. Adrenal vein sampling (AVS) is considered the most reliable method for locating excess hormone secretion. However, it is expensive, invasive and difficult to perform, requiring an experienced radiologist specialized in adrenal vein catheterization. Moreover, the method is limited by different variants of the vessel course, which may lead to unsuccessful cannulation. Adrenal scintigraphy using the radiotracer 131I-6β-iodomethyl-19-norcholesterol (NP-59) allows for visualization of the adrenal cortex and assessment of its hormonal activity. Furthermore, it is minimally invasive and it is almost always effective, regardless of the skill of the operator. Recent studies have provided evidence that NP-59 adrenal scintigraphy may be proposed as an alternative to AVS, mostly to differentiate types of hyperaldosteronism [15,16,17]. Adrenal cortex scintigraphy with NP-59 is usually a complementary procedure to localize adrenal cortex tissue after biochemical diagnosis of adrenal dysfunction. The main indication is the differentiation between unilateral hormone-producing adenoma and bilateral hyperplasia in patients with hypercortisolism, hyperaldosteronism or hyperandrogenism. The procedure is safe and the contraindications include only pregnancy and active breast-feeding. Inhibition of ACTH secretion through autonomous cortisol production allows the contralateral gland to be visualized. Exclusive or significantly predominant uptake on the tumor side confirms its autonomy in the production of steroid hormones. This also suggests a benign lesion in the adrenal gland.

The aim of the study was to evaluate the usefulness of NP-59 SPECT scintigraphy in the imaging diagnostics of MACS.

## 2. Materials and Methods

This retrospective study included twenty consecutive hypertensive patients with benign bilateral adrenal incidentalomas and MACS from 2023 to 2025 in whom primary aldosteronism and pheochromocytoma had been excluded and DHEA-S levels were normal. According to the European Society of Endocrinology guidelines, MACS was diagnosed in patients with two DSTs > 1.8 µd/dL, ACTH < 10 pg/mL, and without obvious, overt signs and symptoms of Cushing’s syndrome. Conditions that alter DST results were excluded. Information was collected on the following clinical and laboratory data: age, gender, body mass index (BMI), DST, diurnal urine free cortisol (UFC), ACTH, late night salivary cortisol (LNSC), midnight serum cortisol (MSC), significant comorbidities (e.g., diabetes, cardiovascular diseases, etc.), tumor sizes and densities (Table 1).

**Figure 1:**
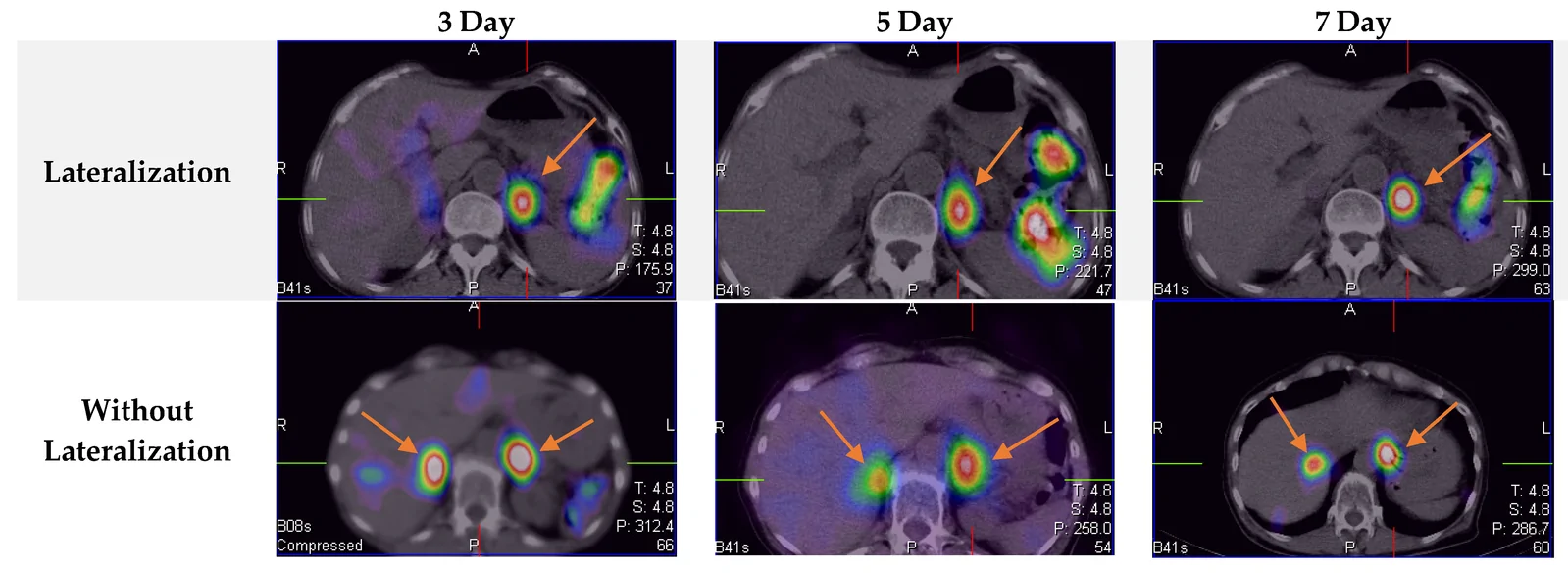
Scintigraphic images of the adrenal glands with and without cortisol lateralization at 3, 5, 7 days after NP-59 administration.

Patients were divided into two groups. The first group consisted of patients with arterial hypertension without other comorbidities (hypertension only, HO) (70%) and the second group included hypertensive patients with comorbidities (hypertension with comorbidities, HC) (30%): 1 with ischemic heart disease (post STEMI), 2 with stroke, 1 with end-stage chronic renal disease requiring dialysis therapy, 1 with poorly controlled rheumatoid arthritis, and 1 with poorly controlled asthma. Adrenal metabolic activity was assessed using the SPECT/CT system after intravenous injection of NP-59. Prior to NP-59 administration, patients received a saturated solution of potassium iodide to block thyroid uptake. Adrenocortical scintigraphy was performed 3, 5, and 7 days after administration of NP-59 (22.8 ± 0.5 MBq). SPECT scanning was performed using a 128 × 128 pixel matrix with a diameter of 4.8 mm and a total of 64 projections over 360° at a time of 40 s/frame. Energy windows were set to 364.0 keV ± 10.0%. After completing the SPECT procedure, CT scans were acquired. Computed tomography was performed using a tube voltage of 130 kVp and the CARE Dose 4D algorithm (Siemens Healthineers, Munich, Germany). In this procedure, the gantry rotation time was 0.6 s, the detector collimation was set to 6 × 2.0 mm, and the pitch factor was 1.8. The CT data were then reconstructed with a slice thickness of 3.0 mm and a field of view of 500 mm. SPECT data were reconstructed using Syngo MI Applications VB20 software (Siemens Healthineers) with the OSCGM and 3D-OSEM algorithms with scatter and attenuation correction based on CT data. Parameters of reconstruction were set to 1 subset and 24 iterations for OSCGM and 1 subset and 50 iterations for 3D-OSEM, and a Gaussian filter with a full width at half maximum of 9.6 mm. According to EANM recommendations and other publications [18,19,20,21,22], lateralization was established when only single adrenal uptake was observed as early as on the third day (Figure 1).

### Statistical Analyses

All data were expressed as mean ± standard deviation, minimum, and maximum. The conformity of the normal distribution was checked using the Shapiro–Wilk test. The differences in the variables between the groups were calculated using the Mann–Whitney U-test for non-parametrical data and t-Student for parametrical data. A *p*-value less than 0.05 was considered statistically significant. Statistical analyses were performed by Statistica 13 by StatSoft Polska.

## 3. Results

All patients were women aged 54–77 (mean = 66). The mean BMI was 27.85 (SD = 4.95), min. 19, max 37. The mean value of the longest diameter of the right tumor was 25.2 mm (SD = 8.24, min = 10, max = 40) and that of the left tumor was 27.5 mm (SD = 8.6 min = 15 max = 49), without significant differences (*p* = 0.9). The mean density according to the Hounsfield scale was 3.7 (SD = 9.17) for the right adrenal tumor and 1.45 (SD = 8.16) for the left (*p* = 0.68).

There were no significant differences in laboratory tests (DST, ACTH, LNSC, MSC, cholesterol) between patients with lateralization and without lateralization in NP-59 SPECT/CT, and between HO group and HC group (Table 2 and Table 3).

**Figure 2:**
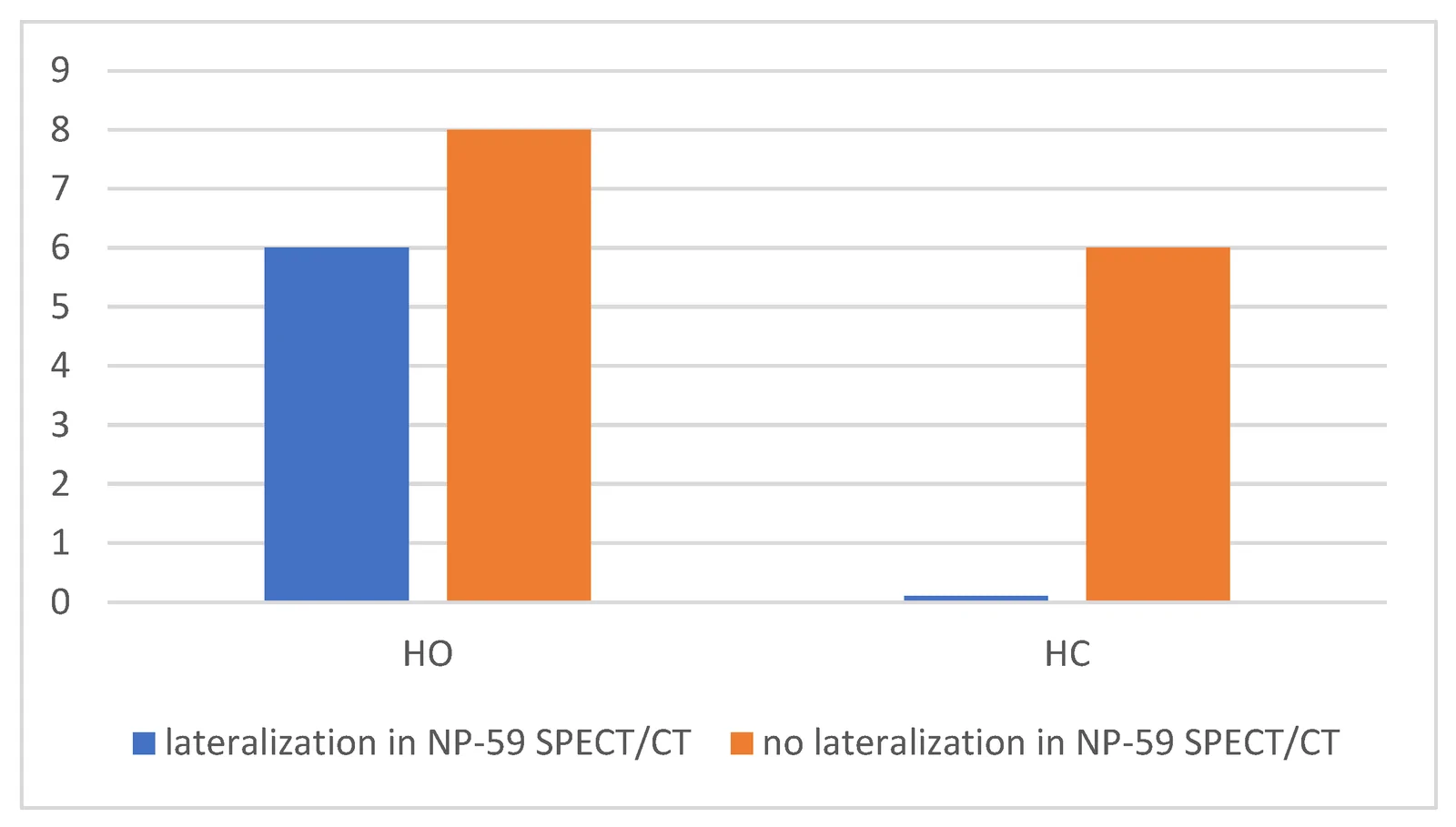
Comparison of the HO group and the HC group in terms of the occurrence of NP-59 lateralization.

Six (30%) patients demonstrated lateralization during NP-59 SPECT scintigraphy (Figure 2) with uptake in the left adrenal gland in two cases and in the right adrenal gland in four cases. All these patients belonged to the HO group and accounted for 43% of this group.

## 4. Discussion

NP-59 was introduced into scintigraphic diagnostics in the late 1970s [23]. As a cholesterol analogue, it accumulates in the adrenal cortex and becomes esterified without being metabolized [24]. NP-59 uptake provides information on both adrenal gland anatomy and function in vivo. Criticism of NP-59 adrenal scintigraphy in lateralization diagnostics has been long-standing. NP-59 scintigraphy without correction based on CT has limited imaging resolution which results in poor detection of small adenomas [25], especially when physiological gallbladder and bowel radiotracer accumulation is superimposed. Small adenomas are more common in primary aldosteronisms than in MACS. The reported sensitivity for detecting an aldosterone-producing adenoma ranging from 47% to 100% depends mainly on the size of the adenoma [26]. NP-59 scintigraphy has been mostly abandoned due to it having several disadvantages, such as poor spatial resolution, less anatomic information and limited availability of the tracer. To improve the spatial resolution, some researchers proposed the use of SPECT. With the simultaneous use of integrated SPECT/CT, the accuracy of the NP-59 scintigraphy has been reportedly improved in a series of studies of diagnostics of primary aldosteronism [27,28,29]. NP-59 conventional planar scintigraphy sensitivity for adenoma in Cushing’s syndrome is reported to be 90–100% with approximately 86% specificity [30,31,32]. So far, there is a lack of data for MACS. MACS is generally more difficult to visualize compared to overt Cushing syndrome. SPECT/CT for MACS may present comparable or even higher accuracy than planar scintigraphy for overt Cushing’s syndrome.

A management strategy based on adrenalectomy, depending on progression to overt Cushing’s syndrome, may be questionable, as the risk of progression from MACS to overt Cushing syndrome is vanishingly low [10,33]. However, despite there being a lower degree of hypercortisolism in comparison with Cushing syndrome, there were no significant differences in comorbidities [12,34]. This suggests that even a mild cortisol secretion increase is sufficient to cause concurrent illnesses [35]. Moreover, 4–12% of patients with adrenal incidentaloma which was non-functioning at first may develop an abnormal DST during subsequent follow-up [36,37,38]. The CHIRACIC study was a prospective study designed to determine whether adrenalectomy, compared with conservative treatment, would benefit patients with unilateral adrenal adenoma, MACS, and hypertension [39]. More than half of the patients were able to discontinue antihypertensive drugs after undergoing adrenalectomy, while this was not achieved in any patient in the conventionally treated group. The authors suggest that these results provide evidence of a causal relationship between MACS and hypertension. Adrenalectomy also significantly improved other prevalent cardio-metabolic elements (type 2 diabetes, dyslipidemia) [40]. In our study, lateralization was found in six of 14 patients from the HO group. The rest of the patients in whom lateralization was not detected should be followed up with, taking into account the possible need for hypertension treatment adjustments and, consequently, normalization of the DST. Interestingly, lateralization was not observed in any of the six patients from the HC group. The reason for this is probably that other diseases, along with uncontrolled hypertension, contribute to the secondary increase in cortisol levels. According to the results of the CHIRACIC study, the success rate of treatment would likely increase significantly after NP-59 SPECT/CT.

It is estimated that 50–65% of patients who undergo adrenalectomy develop adrenal insufficiency [41,42,43]. The incidence of adrenal insufficiency in bilateral adrenal incidentalomas is much lower and accounts for 25.9% [44]. NP-59 SPECT/CT could also be useful for predicting adrenal insufficiency after adrenalectomy, in addition to parameters such as low ACTH levels and lack of response to the CRH test. Reduced or absent uptake in the contralateral adrenal gland on SPECT/CT NP-59 may indicate a high probability of postoperative adrenal insufficiency [45,46]. According to the European Society of Endocrinology guidelines for the management of adrenal incidentalomas, in patients with bilateral hyperplasia or bilateral adenomas and MACS syndrome, individualized treatment options are recommended. The patient’s age, gender, degree of cortisol autonomy, general condition, comorbidities and patient preferences should be taken into account. It is also recommended that all patients with bilateral adrenal incidentalomas undergo the same clinical and hormonal evaluation as those for patients with unilateral adrenal incidentalomas [10]. Currently, there are no specific cut-off values of the DST for which adrenalectomy should be performed. During interpretation, circumstances that may affect DST results should be taken into account. The following pitfalls should be considered: disruption of circadian rhythms, gender/age dependence, test limitations, drug interactions, and preanalytical difficulties. False-positive results may occur with rapid absorption or impaired absorption of dexamethasone due to prolonged intestinal transit time, chronic diarrhea, or celiac disease. Treatment with CYP3A4 inducers (e.g., phenobarbital, carbamazepine, St. John’s wort) and oral estrogens, pregnancy, or chronic active hepatitis increases corticosteroid-binding globulin (CBG), which may increase total cortisol levels [47]. NP-59 SPECT/CT offers clinicians a minimally invasive, reliable alternative to traditional procedures, aiding the identification of unilateral cortisol secretion and guiding treatment decisions such as adrenalectomy versus conservative management.

## 5. Conclusions

Our current study demonstrated lateralization of NP-59 in six hypertensive patients without other comorbidities. Because the NP-59 SPECT/CT, not biochemical tests, differentiated patients, we suspect that patients with lateral cortisol secretion are most likely to develop cortisol-related comorbidities. NP-59 SPECT/CT may provide complementary functional information in selected patients. In nonlateralized cases of MACS, pitfalls should be carefully evaluated and treatment should focus on comorbidities. If these comorbidities occur or worsen, we recommend reassessing the endocrine status with NP-59 SPECT/CT and reconsider the potential benefit of surgical intervention, especially if cortisol lateralization appears. Prospective studies are needed to determine its clinical utility.

## Figures and Tables

**Table 1:** Characteristics of patients. (BMI—body mass index, DST—dexamethasone suppression test, ACTH—adrenocorticotropic hormone, LNSC—late-night salivary cortisol, MSC—midnight serum cortisol, UFC—diurnal urine free cortisol, RTD—right tumor diameter, LTD—left tumor diameter, RTHU—right tumor Hounsfield unit, LTHU—left tumor Hounsfield unit, COM—comorbidities, LAT—lateralization: 0—no R—right, L—left, reum.—poor controlled rheumatoid arthritis, HT—hypertension, MI—post-myocardial infarction).

Age	BMI	DST (<1.8 µd/dL)	ACTH (10–60 pg/mL)	LNSC (<1.2 ng/mL)	MSC (<5.4 ug/dL)	UFC (10–100 µd/24 h)	RTD	LTD	RTHU	LTHU	COM	LAT
70	24	4.1	1.45	0.09	8.32	27.1	25	28	−2	11	HT + dialysis	0
62	28	3.2	3.5	0.092	9.62	42.0	20	33	7	7	HT	0
66	30	7.5	4.75	0.12	7.8	36.9	30	32	11	12	HT + reum.	0
73	26	2.9	7	0.14	4.8	31.1	31	19	7	9	HT + stroke	0
59	30	2.96	10	0.19	3.7	17	30	17	10	3	HT	0
68	21	4.31	4.4	0.09	6.84	15.5	19	39	−10	−15	HT + stroke	0
77	25	2.84	9.4	0.097	4.85	25.6	20	31	0	0	HT	0
69	23	2.76	4.8	0.06	6.86	13.2	40	24	−9	−10	HT	R
61	34	3.2	10	0.091	3.2	41.3	28	15	−3	−5	HT	0
54	34	3.44	6.3	0.058	2.31	16.2	16	16	−8	−8	HT	0
68	32	3.3	8	0.41	4.4	15.9	33	28	12	8	HT + MI	0
67	34	2.34	9	0.099	3.52	36.2	29	34	4	4	HT	L
66	24	2.38	8	0.099	4.45	22.7	37	26	8	−2	HT	0
66	19	6.1	6.7	0.08	6.7	24.9	25	27	10	−2	HT + asthma	0
67	31	2.04	8.2	0.078	5.88	16.8	17	23	19	15	HT	0
69	37	15	3	0.56	16.32	21.2	18	32	12	10	HT	L
55	30	4.3	10	0.11	5.0	14	10	49	20	−3	HT	L
68	27	2.78	9.3	0.13	4.73	19.3	36	32	−5	−2	HT	0
71	26	4.1	5.4	0.092	3.5	17.1	26	15	−4	4	HT	R
65	22	5.99	5.4	0.058	4.18	20.76	14	31	−5	−7	HT	L

**Table 2:** Laboratory test comparison between patients with lateralization and without lateralization in NP-59 SPECT/CT (DST—dexamethasone suppression test, ACTH—adrenocorticotropic hormone, LNSC—late-night salivary cortisol, MSC—midnight serum cortisol, UFC—diurnal urine free cortisol).

Tests	Lateralization in NP-59 SPECT/CT			No Lateralization in NP-59 SPECT/CT			*p* Value
	Mean (SD)	Min.	Max.	Mean (SD)	Min.	Max.	
DST	5.7 (4.7)	2.34	15	3.6 (1.48)	2.04	7.5	0.51
ACTH	6.26 (2.67)	3	10	6.92 (2.6)	1.45	10	0.65
LNSC	0.215 (0.21)	0.058	0.56	0.11 (0.04)	0.058	0.19	0.84
MSC	6.56 (5.46)	3.5	16.3	4.94 (2.07)	2.3	9.6	0.90
UFC	20.4 (12)	13.2	36.2	25.16 (9.5)	15.5	42.0	0.26

**Table 3:** Laboratory test comparison between group with other comorbidities vs. group with hypertension (DST—dexamethasone suppression test, LNSC—late night salivary cortisol, ACTH—adrenocorticotropic hormone, MSC—midnight serum cortisol, UFC—diurnal urine free cortisol, HO—hypertension only, HC—hypertension with comorbidities).

Tests	HC Group			HO Group			*p* Value
	Mean (SD)	Min.	Max.	Mean (SD)	Min.	Max.	
DST	4.7 (1.76)	2.9	7.5	4.9 (4.0)	2.38	15	0.08
ACTH	5.38 (2.36)	1.45	8	7.48 (2.57)	3	11	0.11
LNSC	0.118 (0.45)	0.08	0.19	0.17 (0.19)	0.058	0.56	0.86
MSC	6.47(1.57)	4.4	8.32	5.76 (3.95)	2.31	16.32	0.15
UFC	25.23 (8.43)	15.5	36.9	21.12 (4.31)	17	25.6	0.65

## Data Availability

The raw data supporting the conclusions of this article will be made available by the authors on request.

## Abbreviations

HO	Hypertension only
HC	Hypertension with comorbidities
MACS	Mild autonomous cortisol secretion
HPA	Hypothalamus–pituitary–adrenal axis
DST	Dexamethasone suppression test
AVS	Adrenal venous sampling
NP-59	131I-6β-iodomethyl-19-norcholesterol
BMI	Body mass index
UFC	Urine free cortisol
LNSC	Late-night salivary cortisol
MSC	Midnight serum cortisol
